# A Novel Case of Robotic Retroperitoneal Lymph Node Dissection for Metastatic Sertoli Cell Tumour: A Case Report

**DOI:** 10.7759/cureus.96686

**Published:** 2025-11-12

**Authors:** Pranav Patel, Stuart Henderson, Kamran Haq, Ben Challacombe

**Affiliations:** 1 Surgery, Southampton General Hospital NHS Foundation Trust, Southampton, GBR; 2 Urology, Guy's and St Thomas' NHS Foundation Trust, London, GBR

**Keywords:** neuro-endocrine tumour, onco-surgery, retroperitoneal lymph node dissection, robotic surgery, sertoli cell tumor

## Abstract

Sertoli cell tumours of the testes (SCTT) are a rare subgroup of sex cord stromal tumours. This report describes a novel use of robotic retroperitoneal lymph node dissection (RPLND) in a rare case of metastatic disease that posed significant diagnostic challenges. A 60-year-old male presented with a left testicular mass; post-orchidectomy histological review confirmed high-risk Sertoli cell tumour. A subsequent PET-CT identified metastatic para-aortic lymph nodes; an incidental finding of a non-functioning paraganglioma was also noted. The patient underwent a successful robotic RPLND, performed using the DaVinci Xi robotic platform. Postoperative recovery was uneventful, and the patient was discharged on postoperative day two. Histology confirmed metastasis in two out of 10 lymph nodes. This report highlights the potential of robotic RPLND as a viable treatment course, with its success emphasising the critical role of multidisciplinary team collaboration. While we describe a novel approach, further studies and long-term oncological outcomes in larger patient cohorts are necessary to establish definitive treatment guidelines.

## Introduction

Sertoli cell tumours of the testes (SCTT) are a rare subset of sex cord-stromal testicular tumours, accounting for approximately 1% of all testicular neoplasms [1]. The majority are benign and typically present in younger patients. In a meta-analysis of 435 cases of malignant SCTT, 50 cases (11.5%) demonstrated metastasis, with the retroperitoneal lymph nodes being the most frequent site (76%) [2]. Due to the rarity of the disease, no formal treatment guidelines exist, and consensus on management is lacking. Evidence for systemic therapies is limited to a few isolated reports; for example, Shang et al. described a patient with metastatic malignant SCTT who responded well to sintilimab and apatinib, followed by an R0 retroperitoneal lymph node dissection (RPLND) [3]. While promising, this was part of a retrospective cohort study where only one of four metastatic sex cord-stromal tumours achieved an objective response. To date, there are no published clinical trials or larger studies demonstrating the efficacy of chemotherapy or radiotherapy for SCTT.

We describe a case of retroperitoneal metastatic disease presenting with diagnostic uncertainty in which robotic RPLND was successfully performed for the first time in this context. Robotic RPLND has been shown to decrease blood loss and shorten hospital stays, without a significant difference in recurrence rates compared to open RPLND, as demonstrated in a propensity-matched analysis by Chavarragia et al. [4].

## Case presentation

**Figure 1:**
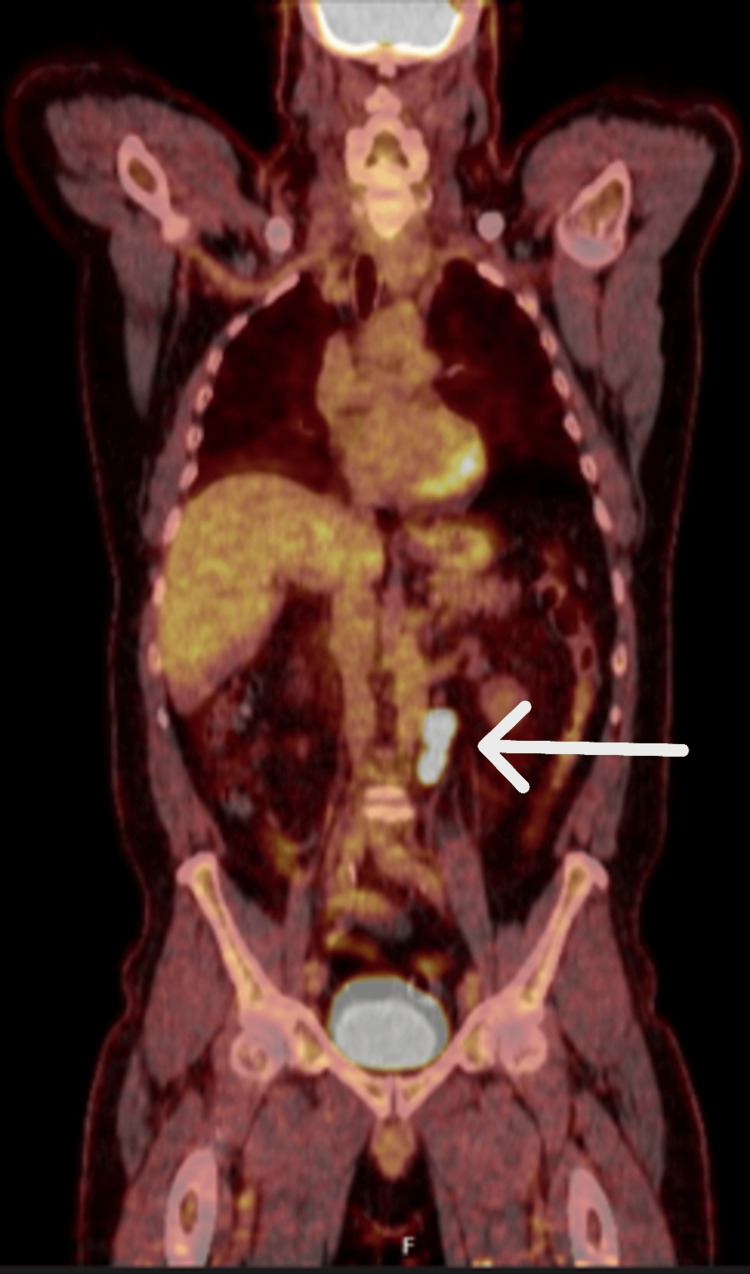
Fluorodeoxyglucose PET-CT showing increased uptake in in the para-aortal retroperitoneal region (arrow) PET-CT: positron emission tomography-computed tomography

A 60-year-old male with a background of hypertension and benign prostatic hyperplasia presented to a regional centre with a left-sided testicular mass. He underwent a left radical orchidectomy with subsequent histology and immunohistochemistry showing a 60 x 57 x 25 mm tumour with a Ki67 index up to 30%, increased mitosis, and high-grade cytological atypia suggesting a high-risk SCTT. This represents an atypical age for presentation, as Yuh et al. reported a median age of 36 years among 19 SCTT patients [5]. A staging PET-CT with fluorodeoxyglucose radiotracer identified two avid para-aortic lymph nodes (Figure 1). The patient was referred to our tertiary centre for further management.

Re-examination of histology and immunohistochemistry confirmed a 60 × 57 × 25 mm Sertoli cell tumour, showing WT1 (+), CD30 (-), OCT3/4 (-), inhibin (-), AE1/AE3 (+), vimentin (focal +), synaptophysin (+), calretinin (-), AFP (-), chromogranin (-), S100 (-), beta-catenin (nuclear +), with a Ki-67 proliferation index of up to 30%. These features are associated with poor prognosis, and their presence supports classification as a high-risk malignancy.

**Figure 2:**
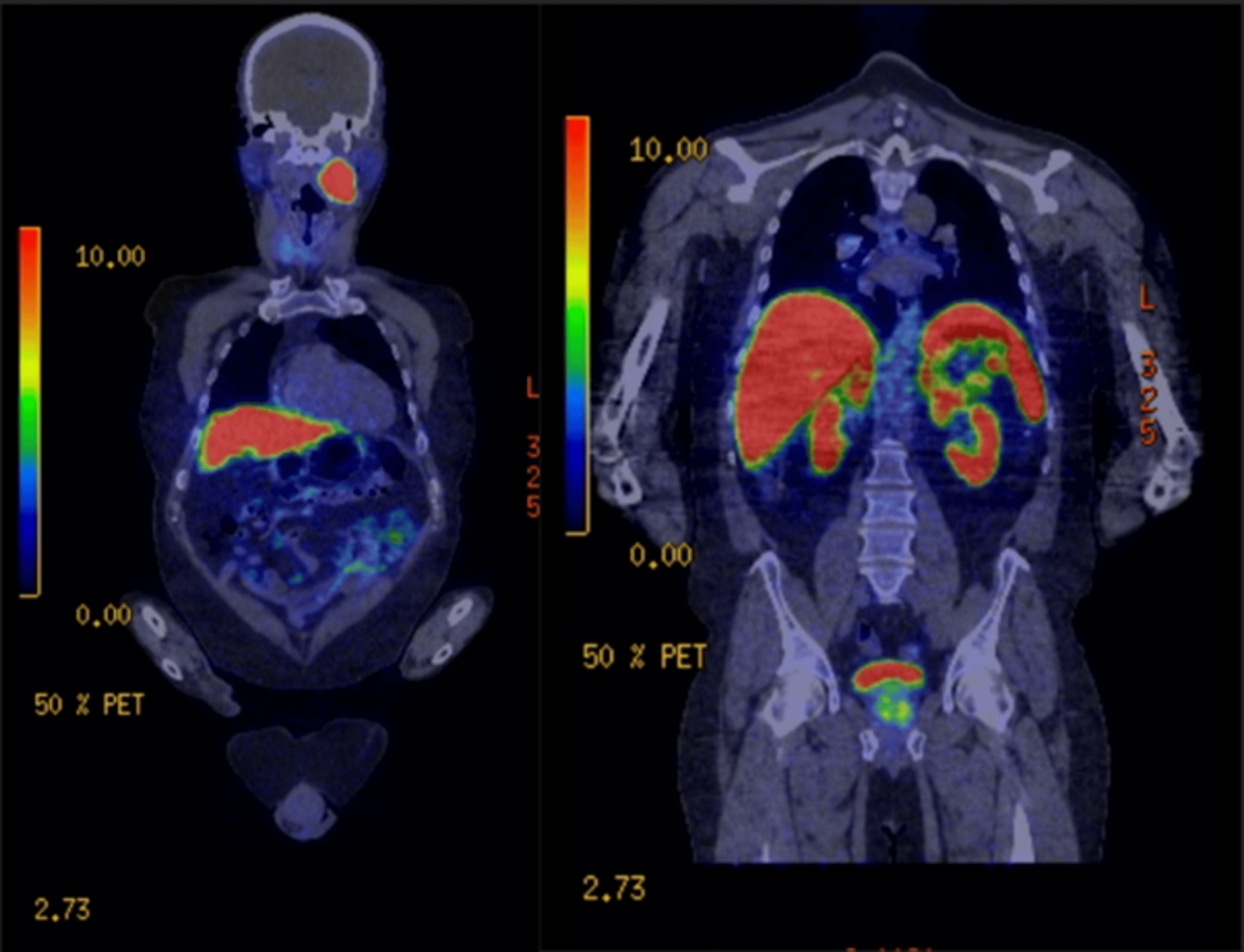
DOTA-TATE PET-CT showing increased uptake in the left neck (left panel), but no increased uptake in the retroperitoneal para-aortal area (right panel)

A CT of the neck, thorax, abdomen, and pelvis revealed an incidental lesion in the neck, raising suspicion for a possible additional metastasis. Further investigation with PET-CT using a Gallium 68 1,4,7,10-tetraazacyclododecane-tetraacetic acid tyr3-octreotate (DOTA-TATE) radiotracer showed increased uptake in the left carotid sheath and parapharyngeal space, with no increased uptake in the retroperitoneal space, suggesting an unrelated primary (Figure 2). Increased DOTA-TATE uptake is associated with neuroendocrine tumours, raising the possibility of a paraganglioma. However, negative blood metanephrines on testing indicated a non-functioning paraganglioma and the patient was referred to Head and Neck specialists for further evaluation, but no further intervention was carried out in the acute follow-up period (three weeks). Further case review by the multidisciplinary team (MDT) recommended robotic RPLND to resect the retroperitoneal disease.

Procedure

**Figure 3:**
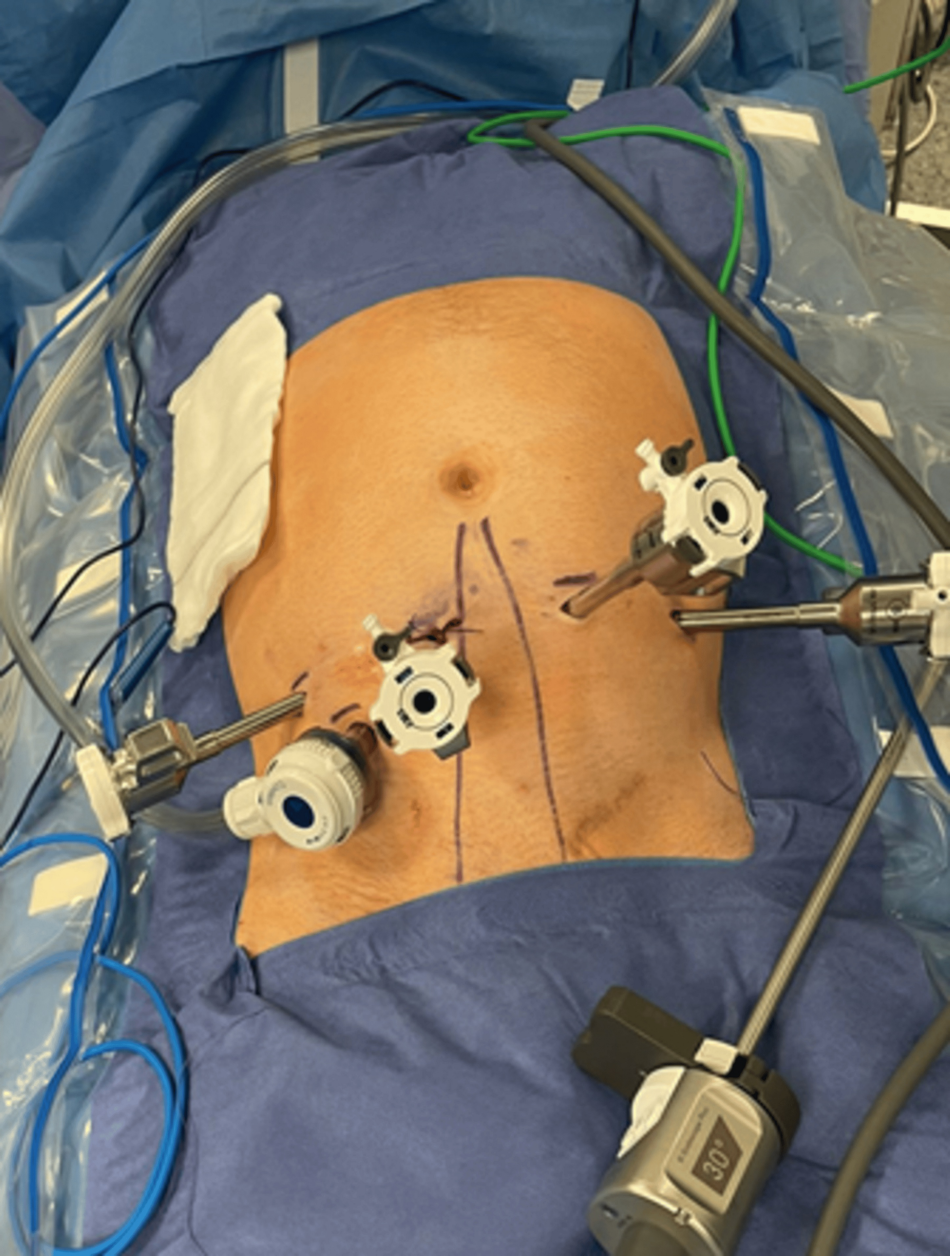
Standard robotic port placement

The procedure was performed by a high-volume robotic surgeon with extensive experience in RPLND, assisted by a senior fellow and an experienced surgical care practitioner at the bedside. The DaVinci Xi robotic platform was used. Four robotic ports and a single 12-mm assistant port were placed (Figure 3). The patient was positioned in the Trendelenburg position.

Access to the retroperitoneum was gained via mobilisation of the mesenteric root, with internal retraction sutures used to create space for the dissection. Initial dissection focussed on the identification of landmarks, including the aortic bifurcation and the inferior vena cava. Dissection commenced in the aorto-caval region and was then extended toward the para-aortic area. Performing the initial dissection in a non-diseased area allowed extension of a safe plane over to the diseased area, where the distinction between planes was more challenging. The inferior mesenteric artery was identified and preserved. Lateral extension of the field enabled visualisation of the left ureter.

Dissection was completed in both the para-aortic and inter-aorto-caval regions. The boundaries of the template were as follows: superior - renal vessels, inferior - aortic bifurcation, lateral - left ureter, medial - medial aspect of the inferior vena cava. The enhanced view and dexterity of the robotic platform allowed for identification and preservation of the sympathetic chain, as well as lymphatics and lumbar vessels.

During dissection in the aorto-caval region, there was injury to a small lumbar vein, which was controlled using a robotic clip. Examination of the post-resection field showed good clearance posterior to the great vessels. The gonadal vein was dissected down to the deep inguinal ring and divided; the remnant of the cord was also resected for analysis. Haemostasis was then achieved via diathermy and the use of haemostatic agents, including VeraSeal and SURGICELL Fibrillar. We placed a 19Fr JP drain via the lateral robotic port site. 

Postoperatively, the patient was placed on a low-fat diet for two weeks to minimise the risk of chyle leak. Drain and catheter were removed on postoperative day one, and the patient was discharged on day two. A routine follow-up at three weeks in our testicular cancer clinic revealed no complications. Histological analysis of the para-aortic region confirmed metastatic Sertoli cell tumour (largest diameter of 55 mm) in 2/10 lymph nodes. The aorto-caval nodal yield was 12, with no evidence of tumour (total 2/22). Examination of the spermatic cord showed no evidence of tumour.

## Discussion

We described a rare presentation of SCTT, with a confounding radiological finding of a non-secreting paraganglioma. With limited options and scarce data on the efficacy of systemic anti-cancer therapy (SACT), a primary robotic RPLND was successfully undertaken, confirming the presence of viable metastatic disease. Ongoing surveillance will be conducted through regular cross-sectional imaging (CT). The involvement of an MDT was key to our approach, enabling comprehensive evaluation of both chemotherapy options and the complex radiological findings to ensure safe and effective decision-making. Robotic RPLND represents an alternative to SACT, but adequate surgical clearance of the diseased regions remains key. It remains a technically challenging procedure with a potential for major complications and should generally be performed in high-volume centres with expertise in the surgical management of metastatic testicular cancer. In cases of incomplete clearance, salvage options would be extremely limited, given the uncertain responsiveness of Sertoli cell tumours to currently available SACT.

In a meta-analysis, Grogg et al. reported the treatment of 28 metastatic cases (out of 50): of the 12 patients who received surgery, five showed remission, of whom four had RPLND [2]. However, due to the exceedingly small sample size and high rates of variability, it is not possible to extrapolate the effectiveness of RPLND for the entire patient population. Yuh et al. [5] observed that the prognosis for patients with metastatic SCTT is poor, and due to the limited efficacy of radiotherapy and chemotherapy, therapeutic RPLND remains the mainstay of treatment. Mosharafa et al. [6] also looked specifically at the curative role of RPLND in sex cord-stromal cell tumours. Their study also had a small sample size, with only 17 patients, four of whom had confirmed SCTT. Of them, only the two patients with Stage I (T1-4, N0, M0) survived at 63- and nine-month follow-up, respectively. The other patients had Stage IIA (any T, N2, M0: lymph nodes >2 cm but not >5 cm, or multiple lymph nodes with any one mass measuring >2 cm but not >5 cm), and Stage IIIA disease (any T, any N, M1a: nonregional lymph node or pulmonary metastasis). They both died of the disease at nine and 69 months, respectively.

The literature on the treatment of metastatic Sertoli cell tumours remains sparse; however, the limited efficacy of systemic treatments is a common theme. Surgical resection in appropriately selected cases, therefore, offers an alternative means of treatment for metastatic disease. RPLND is a well-established, safe, and feasible procedure in the management of more common forms of testicular cancer, making its application to high-risk sex cord stromal tumours seem logical. While the retroperitoneal lymph nodes are the most common site of metastasis (76%) [2], other metastatic sites have also been reported, suggesting that prophylactic RPLND may not be effective in all settings. The same meta-analysis, which evaluated 435 patients, identified several key risk factors for metastasis: age over 27.5 years (odds ratio (OR): 15.8), tumour necrosis (OR: 102), extension into the spermatic cord (OR: 20), and a high mitotic index (OR: 37.1) [2]. Our patient was older, and the tumour had a high mitotic index, indicating a greater risk.

Another method of identifying risk may be through genetic sequencing; one study of 22 metastatic SCTTs found that around 50% had a CTNNB1 mutation [7], suggesting a mutation to screen for which has low sensitivity. Our centre also performed fluorescent in situ hybridisation to detect an EWSR1 22q12 chromosomal rearrangement, which was negative. This rearrangement, initially described in Ewing’s sarcoma, has also been reported in highly aggressive sex cord stromal tumours [8]. Larger studies are required to better identify Stage I patients with significant risk factors who might benefit from prophylactic RPLND.

## Conclusions

We discussed a unique case of metastatic SCTT, with a confounding radiological finding of a non-secreting paraganglioma. This was treated via robotic RPLND with resection of the para-aortic and inter-aortocaval regions. The patient was discharged on day two postoperatively and has not experienced any surgical complications to date. Histology revealed a viable tumour in the 2/22 nodes that were avid on preoperative PET-CT. To our knowledge, this is the first reported application of minimally invasive (robotic) RPLND to treat metastatic Sertoli cell cancer. Careful patient selection remains paramount, and given the limited evidence in the field, multidisciplinary team involvement is essential. In the absence of proven effective systemic therapies, we have shown that RPLND offers a safe, novel, and viable treatment alternative. Longer-term oncological outcome data are required to inform the development of robust guidelines for this patient population.
